# Development and Application of a Wireless Sensor for Space Charge Density Measurement in an Ultra-High-Voltage, Direct-Current Environment

**DOI:** 10.3390/s16101743

**Published:** 2016-10-20

**Authors:** Encheng Xin, Yong Ju, Haiwen Yuan

**Affiliations:** 1 School of Automation Science and Electrical Engineering, Beihang University, Beijing 100191, China; enchengfamily@buaa.edu.cn; 2 China Electrical Power Research Institute, Beijing 100192, China; juyong@epri.sgcc.com.cn

**Keywords:** ultra high-voltage direct-current (UHVDC), space charge density, energy consumption, wireless communication, Zigbee

## Abstract

A space charge density wireless measurement system based on the idea of distributed measurement is proposed for collecting and monitoring the space charge density in an ultra-high-voltage direct-current (UHVDC) environment. The proposed system architecture is composed of a number of wireless nodes connected with space charge density sensors and a base station. The space charge density sensor based on atmospheric ion counter method is elaborated and developed, and the ARM microprocessor and Zigbee radio frequency module are applied. The wireless network communication quality and the relationship between energy consumption and transmission distance in the complicated electromagnetic environment is tested. Based on the experimental results, the proposed measurement system demonstrates that it can adapt to the complex electromagnetic environment under the UHVDC transmission lines and can accurately measure the space charge density.

## 1. Introduction

The electromagnetic environment under UHVDC transmission lines contains several inter-related electrical parameters, which are the electric field, the ion current density, the space charge density, the radio interference, the audible noise, and the magnetic field [1,2]. With the development of UHVDC transmission and transmission capacity enlargement, the generation of corona will be inevitable. Air molecules surrounding the conductor surfaces of the lines will be ionized, giving rise to positive and negative space charges, which are distributed in the space between the transmission lines and the ground [3]. Charged particles are formed by capturing charged clusters. The charged particles in the corona area will affect the space electric field distribution of ionization zone and then affect the corona discharge. The charged particles in the corona ion migration region, as space charge, will also affect the ion flow field. The effect that particulate matter, which is in the form of space charge to impact the corona discharge and space electric field, is referred to as particulate matter space charge effect. The space charge effect of charged particles is one of the important factors to affect the DC corona discharge and high-voltage DC transmission line electromagnetic environment. This means it is important for the DC power transmission project to research the interaction between space charge and the electromagnetic environment of UHVDC transmission lines [4,5,6,7].

A sensor is a key part of a space charge density measurement system. There is much literature about the measurement of the charge density in air. In [8], German researcher Ebert used two coaxial brass pipes as electrodes, and air was aspirated into the device by an impeller in 1901. In [9], Misakian from the National Bureau of Standards of America measured the charge density with a rectangular cuboid aspirator-type ion counter arranged in a cavity below the grounded electrode in 1986. However, the device was not used at other locations in the field, or measured the charge density under the UHVDC lines. In [10], Rossner and Singer from Technical University of Hamburg-Harburg also applied an aspirator-type apparatus to measure charged particles in a diameter of 1.5–10 μm in 1989. Relative error of measured results might be 20% when compared with an optical particle counter, which shows the difficulty of measurement of charge and particle density. In [11], Kulon from Brunel University applied phase Doppler anemometry with a laser 500 nm in wavelength to measure the charge distribution of 0.7–1.9 μm particles in a parallel plate field in 2002. However, it may be difficult to popularise this expensive laboratory technique to measure the charge densities under outdoor transmission lines.

In this paper, relying on the wireless communication technologies, a space charge density measuring sensor is put forward with an ion counter method. The main goal of this study is to develop a wireless sensor network (WSN) aiming for the reliable and flexible measurement of the space charge density under UHVDC transmission lines. This paper is organized as follows. In Section 2, the measurement system framework is described. In Section 3, the measurement principle and design of the space charge density measurement system is proposed. In Section 4, wireless network selection and communication quality evaluation is elaborated. Finally, a conclusion section ends this paper.

## 2. Space Charge Density Measurement System Framework

**Figure 1:**
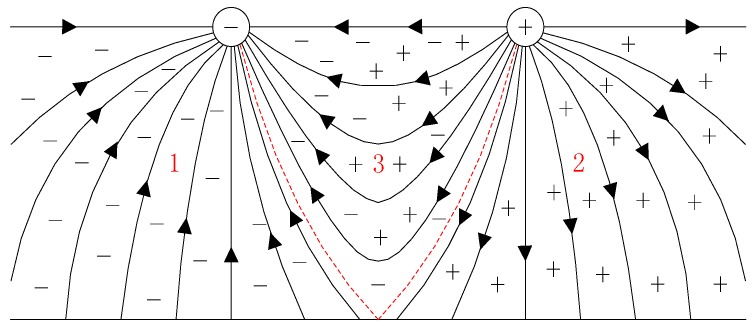
Space charge distribution around direct current transmission lines.

The generation of space charge is mainly caused by corona and dose directional movement between direct current conductor and earth. Due to the polarity of the wire being different, the formation mechanism and the movement direction of space charge are also slightly different. There are three different parts formed under UHVDC transmission lines as shown in Figure 1. In this figure, the earth’s fair-weather electric field has been ignored. They are negative ion area 1, positive ion area 2 and ion mixing zone 3.

**Figure 2:**
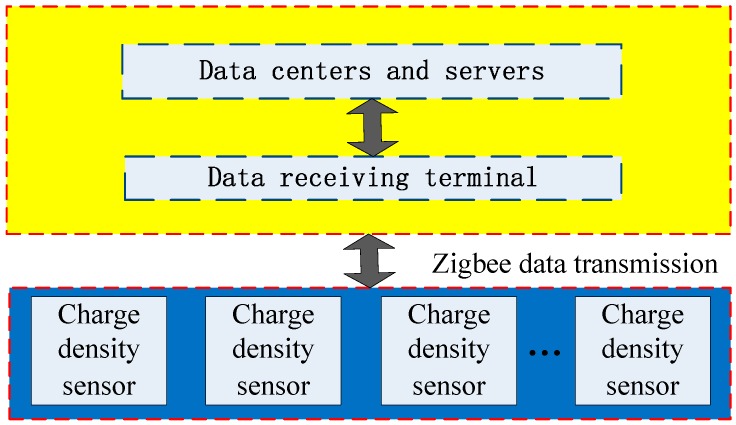
Structure of the distributed measurement system.

The space charge density under UHVDC transmission lines is measured at different positions through a number of charge density sensors. The result comes from multiple sensors which can imply the distribution of the space charge density. The framework of the space charge density measurement is shown in Figure 2. The wireless sensor is used to measure the space charge density. Data collected is sent to the remote node by Zigbee module. The remote node connected with the data centers and servers. The value of the space charge density is displayed on the screen and stored in the database. As a result, the distribution of the space charge density under UHVDC transmission lines can be achieved through multiple sensors system usage.

## 3. Design of the Space Charge Density Measurement System

### 3.1. Space Charge Density Sensor Design and Working Principle

**Figure 3:**
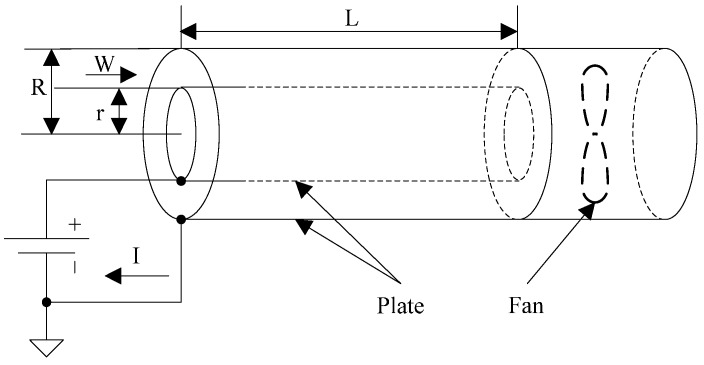
Sensor operation principle.

The sensor realizes the measurement of space charge density using the ion counter method, and its structure design is shown in Figure 3. The sensor consists of plates and a fan with a constant speed. The working principle of the sensor is that charged ions in the atmosphere pass the plate electrode under the action of fan, and then current is formed on the plate by ions movement under the action of the electric field force between the plate to plate. By measuring the current through the plate and gas flow within a certain time, the charge contained in gas and gas volume through the plate and the charge density will be obtained. The calculation formula of space charge density is given by

(1) $$\rho =\frac{Q}{V}=\frac{\int_{t_{0}}^{t}I(t)dt}{\int_{t_{0}}^{t}\pi \left(R^{2}-r^{2}\right)W(t)dt}$$

where *ρ* means the space charge density; *Q* means the charge arrived at plate in a particular time period; *V* means the gas volume flow between plates; *I* means the current flows through the plate; *R* means the radius of the outer plate; *r* means the radius of the inner plate; and *W* means the flow velocity in the device as controlled by the fan.

Ion movement speed is related to mobility. The mobility of charged ions means the final velocity of the unit field strength charged ion movement. The speed is related to several factors such as the temperature, pressure, particle size of the ion.

When the applied voltage and the gas velocity between the plates are different, the number of charged particles which will reach the plate is also different. The scope of the charged particles which reach the plate can be expressed with critical mobility. The expression is given as

(2) $$k_{c}=\frac{M\varepsilon_{0}}{CU}$$

where *M* is the gas flow per unit time, *ε*_0_ is the vacuum dielectric constant, *C* is the plate capacitor, *U* is the voltage between plates. For coaxial cylinder type plate, critical mobility of charged particle counter is

(3) $$k_{c}=\frac{W\left(R^{2}-r^{2}\right)\ln\left(\frac{R}{r}\right)}{2UL}$$

where *L* is the length of the plate.

The ion flow field formation by corona discharge contains a variety of ionic components [12]. Ion mobility is associated with ion own characteristic such as quality and volume. The greater the quality and volume is, the smaller the ion mobility is. The positive and negative ions can be divided into two categories: charged particles (large ions) and charged molecular clusters (small ions). Large ion migration velocity is slow ((1–2) × 10^−2^ cm^2^/(V·s)), and small ion migration velocity is quick (1–2 cm^2^/(V·s)).

**Figure 4:**
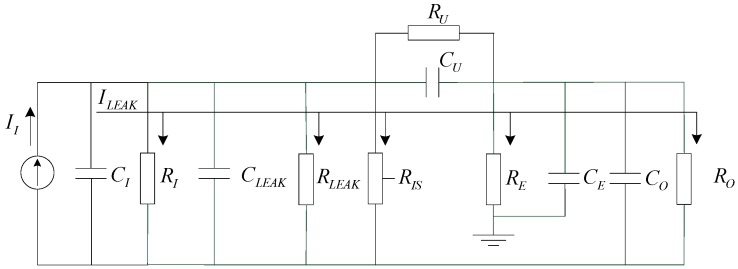
The equivalent model of the space charge density measurement device.

The space charge density measurement device can be equivalent to the combination of a series of resistance and capacitance, and the physical model is shown in Figure 4.

*I_I_* is the current formed by the charged particles hit the plate, *R_I_* is the insulation resistance between the plates, *C_I_* is the equivalent capacitance between the plates, *C_LEAK_* is the stray capacitance, *R_LEAK_* is the insulation resistance of line, *R_IS_* is the leakage resistance of plate bias source module, *R_U_* is the output resistance of the plate bias source, *C_U_* is the output capacitance of the plate bias source, *R_E_* is the equivalent resistance between the charged particle counter and the earth, *C_E_* is the equivalent capacitance between the charged particle counter and the earth, *R_O_* is the input resistance of current measurement circuit, *C_O_* is the input capacitance of current measurement circuit. Leakage current may be produced in *R_I_*, *R_LEAK_*, *R_IS_*, *R_E_* or *R_O_*. In order to ensure the leakage current is small enough, need to make the resistance increased quickly. At the same time, to avoid the influence of stray capacitance on measurements, signal transmission cables should be short.

### 3.2. Design of the Sensor

Sensor design key parameters include the axial incident velocity *W*, the effective length of the tube *L*, inner electrode radius *r* and external electrode radius *R*. In the design of the sensor, there are three main technical problems to be solved. First of all, the sensor has a coaxial structure with an existing edge effect, which may cause distortion of the deflection electric field near the end. It will directly affect the law of motion of ion flow under the action of electric field. Second, this article adopts a ventilation method to provide axial incident ion flow velocity. Its approximate value is equal to the wind speed W. If the wind speed selection is not correct, ions can easily form a turbulent flow within the sensor, and cannot keep constant speed in a level. Lastly, through superposition of an external synthesis electric field, the distortion of the electric field is near the electrode end, interferes with ion movement, and causes the ion current measurement to be inaccurate.

In order to solve the influence of the sensor end effect, the effective length *L* of the sensor needs to be reasonably selected. According to the electric field distribution characteristics, the influence of the end effect in the pipe can be ignored when the effective length *L* >> (*R* − *r*). In order to achieve the ion laminar motion in the sensor, the inner and outer radius of the electrode and wind speed need to be reasonably designed.

Flows with the fluid viscosity may be divided into two types, such as laminar and turbulent, which are determined by the Reynolds number *Re*. The transition from laminar to turbulent occurs at *Re* ≤ 2300. The Reynolds number is defined by

(4) Re=ρVD/η

where *ρ* is fluid density (kg/m^−3^), *V* is the velocity of the air flowing in the pipe (m/s), *D* is the device outer diameter (m), and *η* is the fluid viscosity (Pa·s).

**Figure 5:**
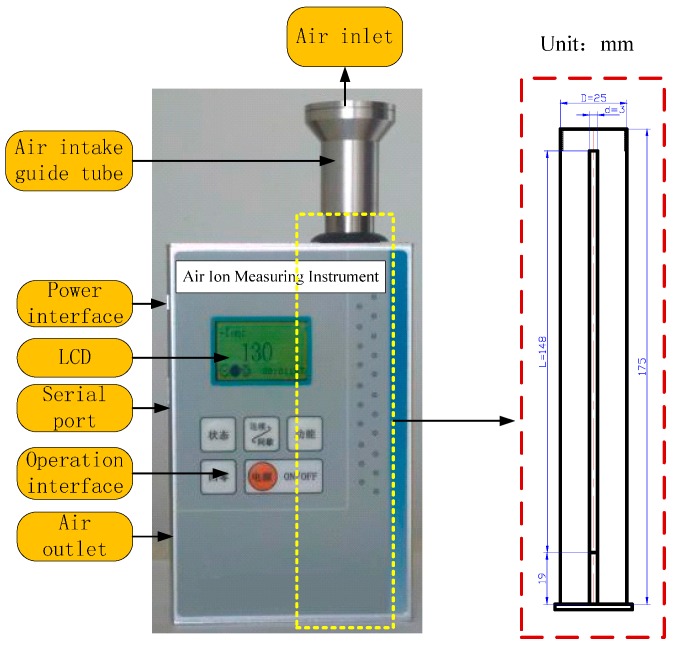
Picture of the sensor.

The structure of the sensor and the main parameters are shown in Figure 5. Its shell is made of aluminum with high surface hardness is used to avoid the accumulation of ion flow under the UHVDC transmission lines. Its external electrode radius *R = D/*2 *=* 12.5 mm, inner electrode radius *r* = *d*/2 = 1.5 mm, effective length *L* = 148 mm, i.e., *L* ≈ 13 (*R* − *r*). The end electric field’s influence on measurement can be ignored. The wind speed *W* in tube is adjustable. When *W* is 1 m/s, *Re* ≈ 167 *<* 2300, can meet the laminar flow conditions in tube. In addition, the voltage between plates is 12 V. Through the calculation of Formula (3), the value of positive ion migration rate is 1.36 cm^2^/(V·s) and the negative ion migration rate is 1.67 cm^2^/(V·s), so we can know that the mentioned ion counter is designed to monitor only small ions.

**Figure 6:**
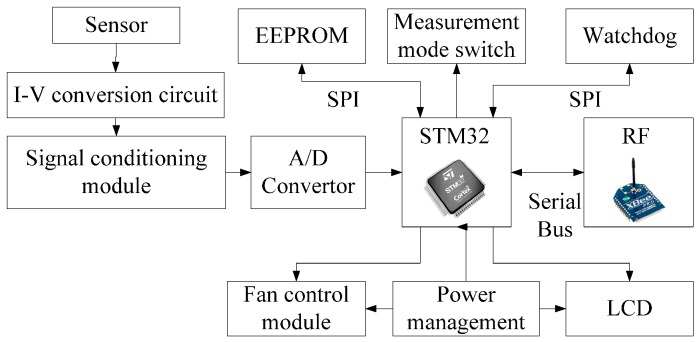
Diagram of the proposed wireless circuit.

**Figure 7:**
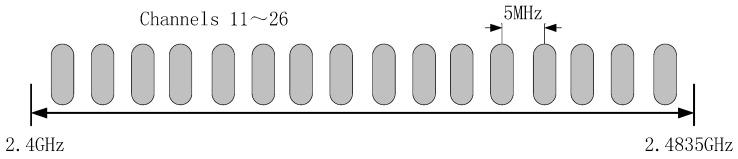
The channels distribution of Zigbee in the 2.4 GHz band.

A diagram of the proposed wireless circuit is presented in Figure 6. As the ion current collected by sensor is too small to collect easily, so the I-V conversion circuit is designed to achieve the conversion of current to voltage. The signal conditioning circuitry is composed of the charge amplifier, the band pass filter and the low pass filter for further processing. The wireless communication system selected for the sensor wireless node is the 2.4 GHz Xbee Pro radio frequency (RF) module based on IEEE 802.15.4 protocol from Digi. The wireless node’s primary function is to acquire and process the output analog signal of the wireless sensor and send the digital signal to the remote node. The IEEE 802.15.4 protocol defines 16 channels whose serial numbers are from 11 to 26 on the frequency band as shown in Figure 7. In actual work, sensor network can choose one of the channels to transmission signal. The RF module can operate from a 2.8 to 3.4 V supply and consume between 45 and 50 mA for receiving and transmitting (R_x_/T_x_) operations. The typical range for the module is 1.5 km line-of sight with 2.0 dB dipole antenna and 90 m indoor.

The STM32F103VE is selected as the microcontroller for the wireless sensor. The processor possesses rich hardware resources. In addition, it has a 3.3 V low voltage. It contains five universal synchronous/asynchronous receivers transmitters (USARTs) and three serial peripheral interfaces (SPI), which are both essential for communicating with the RF module, an electrically erasable programmable read-only memory (EERPOM) nonvolatile module, and a watchdog module. The USARTs not only interface with the RF module but also allow communication with the RS-232 port on a PC. Another valuable feature of the STM32F103VE is that it integrates a real-time clock (RTC) which can provide a clock calendar function for the wireless node. Besides, three 12-bit analog-to-digital converters are embedded into the microcontroller to acquire the analog electric field signal from the sensor. In the signal conditioning module, a low pass filter and an impedance matching circuit are employed to process the amplified signal so that it can be suitable for the analog digital conversion. To minimize the error of the signal conditioning circuit, a high precision operational amplifier with low offset voltage is used. A measurement mode switch unit is used to control the working mode of the wireless sensor (continuous or intermittent). The use of a liquid crystal display (LCD) can facilitate viewing and recording data for operator. The value of the space charge density can be directly obtained through the LCD screen. The power of the LCD, fan control module and frequency module is controlled by the power management unit and can be turned off to the low power mode when the wireless node receives the instruction from the remote computer.

The wireless node acquires the analog signal from the space charge density sensor using the integrated analog-digital (A/D) convertor of the microcontroller. The digital low-pass IIR filter is employed to process the acquired data. The outcome information of the space charge density is calculated and then transmitted to the RF module through the serial bus. It is important to note that the configuration of the wireless node is very flexible. The microcontroller can be easily programmed to connect any space charge density sensor whose calibration data is sent by the PC and saved to the EEPROM.

### 3.3. Software Design of the Measurement System

**Figure 8:**
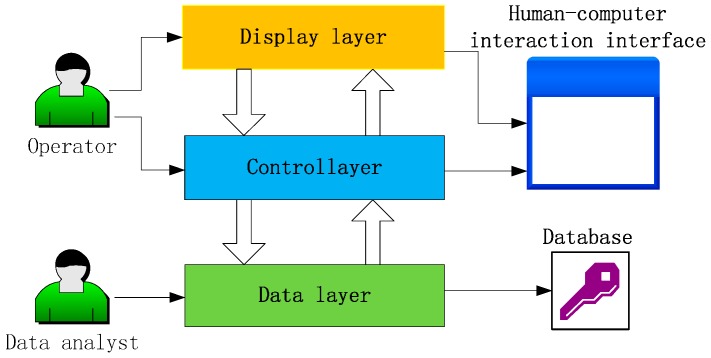
Structure chart of the measurement system software.

Before designing software for the measurement system, demand analysis on the software needs to be considered, because the on-site operator and data analyst have different demands for the software. The on-site operators are required for setting of description of sensor and real-time data collection, as well as real-time surveillance for the node conditions. For data analysts, relevant analyses and processing will be done with the data stored in the database after finishing the measurement. Therefore, software architecture is designed to meet the requirements of different users, as shown in Figure 8. Measuring software consists of display layer, control layer, and data layer.

The display layer, locating at the upper layer, is used for displaying the sensor’s measurement value of space charge density, location information and working conditions; control layer, locating in the middle layer, is the core layer of the three and has much interaction with the remaining two layers, supplying operations to the users so as to realize the real-time control to the space charge density; data layer, locating in the lower layer, realizes the real-time storage of data, in order to facilitate data analysis and processing. Display layer and control layer are composites of interaction surface of human and machine, which is oriented for the on-site operator, while data layer is backed by database, which is oriented for data analysis personnel.

**Figure 9:**
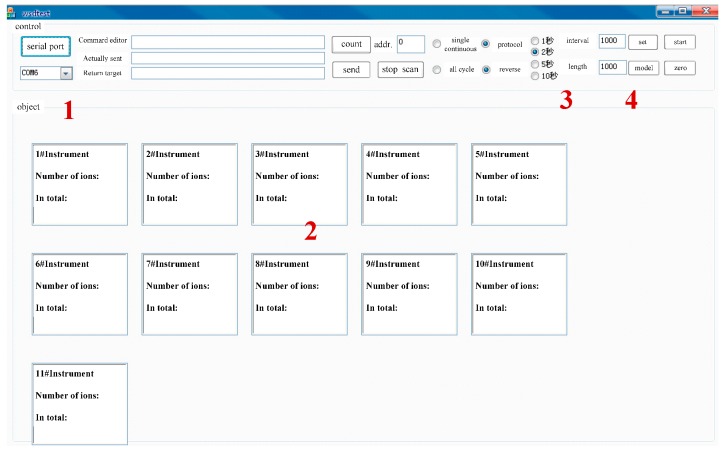
Software interface of the measurement system.

Based on the proposed structure, the software interface of the measurement system is developed as shown in Figure 9. The software interface composites of 4 parts: 1 is the communication serial port, controlling on or off of the serial port; 2 is the data display window, showing location distribution, working state and abnormal information of the sensors; 3 is the data collection interval, showing the selection of data acquisition interval; 4 is the software running control part, controlling whether or not the software is running.

## 4. Implementation of the Distributed Measurement System

### 4.1. Topology of the Wireless Sensor Network

Data transmission uses the Zigbee wireless transmission mode. In according to the equipment’s role, three logical device types are defined for the Zigbee specification. They are, respectively, coordinator, router and end device. The coordinator is the center of the network. It is responsible for establishing and maintaining the network, one and only one in each network; the router is for relay nodes, routing and forwarding data; and the end device, whose function is unitary, is simply to send and receive information.

**Figure 10:**
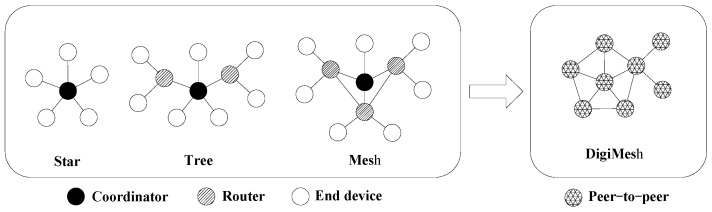
Comparison with different topology of the wireless sensor network.

ZigBee supports three forms of network topology. They are star, tree and mesh topology as shown in Figure 10. The feature of star topology is that data routers between nodes only have one path; the feature of mesh topology is that when one node sends data to another node, the information transfer along the tree path up to the nearest coordinator node, and then passed on to the destination node; the feature of tree topology is that it is a special kind of structure, according to the relay transmission of point to point network structure, and its router can be built and maintained automatically, and has strong self-organization and self-healing function. All the nodes in the network composed of Xbee-Pro modules are defaulted for the peer.

The DigiMesh networking protocol is applied in the wireless sensor network considering the complexity of the operating conditions and wireless transmitting distance under UHVDC transmission lines [13,14]. The DigiMesh network is a simplified mesh network in which all nodes are collaborative and can be either as router or endnode. The characteristic of the network making the expansion of the network is more flexible and increases the robustness in complicated environments under transmission lines where routers may fail due to interference or damage. The topology of the wireless sensor network is shown in Figure 10.

### 4.2. Communication Quality Evaluation

In the course of actual use, there is a condition that the closest node from the main node to help remote nodes to transmit data. The power consumption of these nodes is bound to be a few bigger than others and it will lead to system work time shorter. In order to prolong the lifetime of the whole network, the set of differentiation must be considered.

There are many basic parameters [15,16,17,18,19] used to determine the performance of wireless network system. Packet reception ratio (PRR) is the most intuitionistic communication quality indicators; received signal strength indicator (RSSI) can be used to determine the communication quality; link quality indicator (LQI) shows the energy and quality of the receiving data. PRR, however, needs a long time to send and receive experiments to obtain the data. RSSI and LQI are relatively easier to obtain, and can commutative conversion. The conversion relationship is

(5) RSSI=−(81−(LQI*91)/255)

RSSI value can be read when each data frame is received in the transceiver module of Zigbee, so RSSI data can be more easily used as link quality criteria. However, RSSI is the value difference between actual receiving signal intensity and optimal received power level. That means the higher the absolute value of RSSI is, the stronger the signal decrease is.

## 5. Validation and Experiment

### 5.1. Transmitted Power Consumption Measurement Analysis

Xbee-Pro module support five different emission levels with different operation mode, they are 10 dBm, 12 dBm, 14 dBm, 16 dBm, 18 dBm respectively. To know the relationship between energy consumption and transmission power of the proposed wireless communication unit, a research on energy consumption situation with five different transmission power settings is proceeded under a double circuit experimental line on the same tower in Beijing.

**Figure 11:**
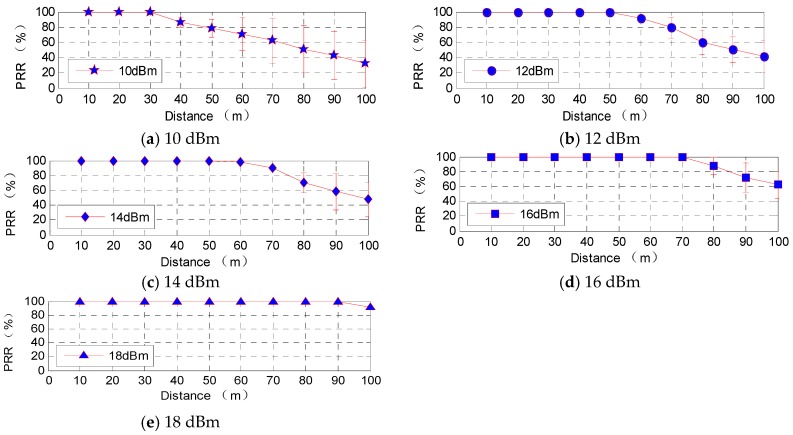
Wireless module data reception with different transmission power levels.

The complicated electromagnetic environment will have an impact on the wireless network communication quality. Therefore, the data on the relationship between power consumption and transmission power of the proposed wireless communication unit were obtained in a complicated environment as shown in Figure 11; PRR and RSSI are not fixed values but fluctuating in a certain range, which therefore cannot be replaced in the test by some instantaneous values, putting them in the category of error connecting performance.

In the complicated electromagnetic environment under UHVDC transmission line, Zigbee wireless communication module can ensure the quality of good communication in 30 m with 10 dBm transmitted power, in 40 m with 12 dBm transmitted power, in 50 m with 14 dBm transmitted power, in 70 m with 16 dBm transmitted power, in 80 m with 18 dBm transmitted power respectively. PRR data from Figure 11 show high transmission power, which to some extent corresponds to good quality of communication. The data can be used as a reference of the transmission power for space charge density measurement system in a complicated electromagnetic environment.

### 5.2. Complex Environment Test

**Figure 12:**
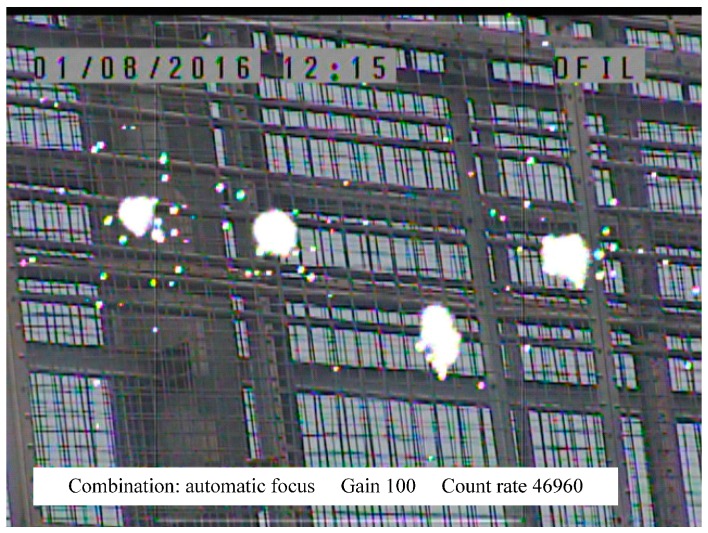
Corona discharge test.

**Figure 13:**
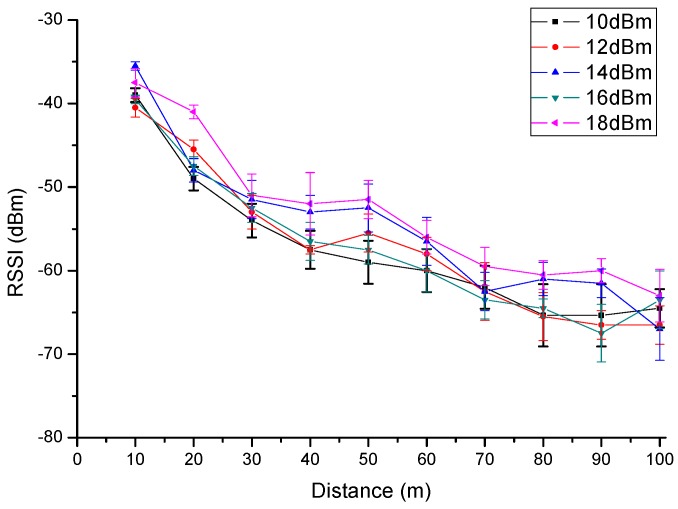
RSSI of 11 channels in the off-power state.

**Figure 14:**
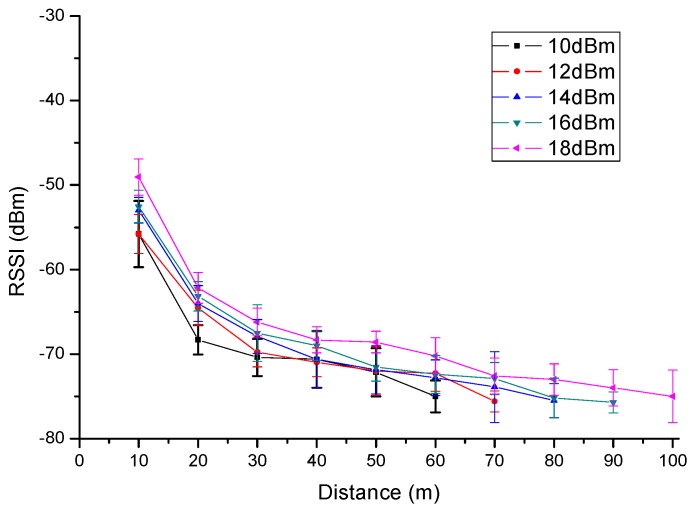
RSSI of 11 channels in the on-power state.

When corona discharge occurs in the UHVDC transmission line, the wireless signal may be affected. The corona discharge from high voltage lines is not continuous. The test with an ultraviolet imager has been shown in Figure 12. This wireless interference mainly comes from negative transmission line. Therefore, the RSSI comparison test is taken in the two conditions of on-power and off-power for the UHVDC transmission line. There are 11 optional channels in the model provided to the users as Table 1 shows. RSSI on different transmission power within 100 m supplied by RF module will be measured as shown in Figure 13 and Figure 14.

Wireless communication unit child nodes were placed under the negative transmission line. From Figure 13, the minimum value of RSSI is −35 dBm and the biggest value is −70 dBm in the off-power state. Correspondingly, the minimum value of RSSI is −47 dBm and the biggest value is −78 dBm in the on-power state in Figure 14. Compared Figure 13 and Figure 14, when test distance is 10 m, the value of RSSI is −35 dBm~−42 dBm in the off-power state and −47 dBm~−60 dBm in the on-power state. Influenced by the environment, part of the transmitted power communication quality become worse with the increase of distance, gradually leading to the RSSI data quantity received being too little with distances greater than 60 m. They are not representative, so the value can be negligible. By contrast, we found that the complex electromagnetic environment will produce certain effects on the wireless communication unit.

Wireless interference field in UHVDC transmission line has a strong intensity in the low frequency stage. It decreases quickly with the increasing frequency. When the frequency is over 10 MHz, interference strength can be neglected. Generally, the wireless interference field intensity that occurred in the corona discharge in UHVDC line is considered 30 MHz at most. The frequency range of this system is around 2.4 GHz, and thus corona discharge in the UHVDC transmission line does not affect the normal work of this system.

### 5.3. Wireless Communication Network Reliability Analysis

**Figure 15:**
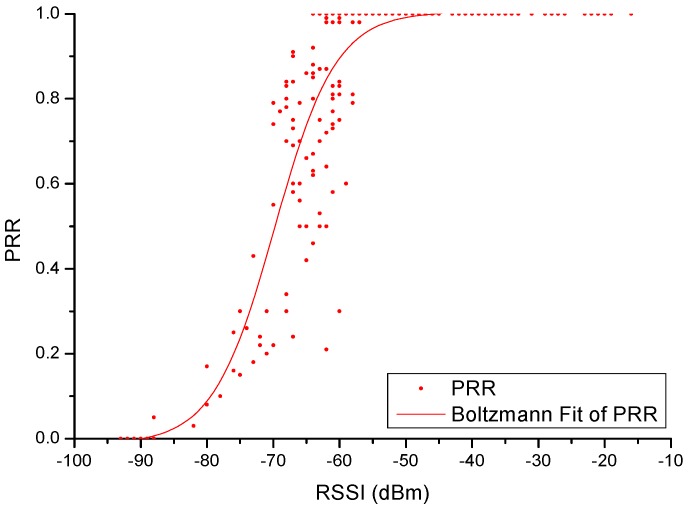
200 times of RSSI and PRR collection.

Xbee-Pro module has different performance in different work environmenta. 200 instances of RSSI and PRR collection are done in each test. The regularity of distribution has been described by a sigmoidal curve defined by a lag phase, subsequent growth, and a final equilibrium phase. It is in conformity with Boltzmann distribution law. The result is as shown in Figure 15.

From Figure 15, when the value is greater than −60 dBm, equipment in the complex work environment can maintain stable communications, and the data receiving rate is above 90%.

### 5.4. Space Charge Density Measurement Test under UHVDC Transmission Line

**Figure 16:**
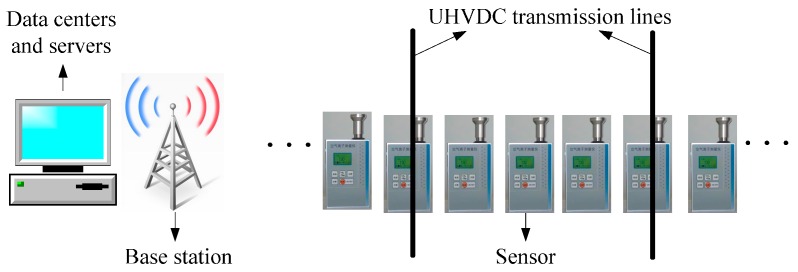
Arrangement diagram of measurement system.

**Figure 17:**
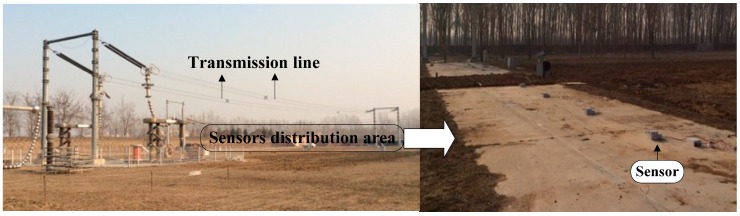
Picture of measurement system used under UHVDC transmission line.

To validate and test the proposed wireless measurement system, the space charge density of 11 different points under the experimental lines was measured. The transmission line is 100 m long. The minimum distance from the ground to conductor is 7 m and the separation distance between the positive pole and the negative pole is 6 m. The origin of the measurement result coordinate was set at the central position of the positive pole and the negative pole. The wireless sensors were arrayed along the direction perpendicular to the transmission lines with the separation from the origin at −15 m, −12 m, −9 m, −6 m, −3 m, 0 m, 3 m, 6 m, 9 m, 12 m, 15 m. The arrangement diagram of the measurement system is shown in Figure 16 and the photo of test base and measurement system is shown in Figure 17.

**Figure 18:**
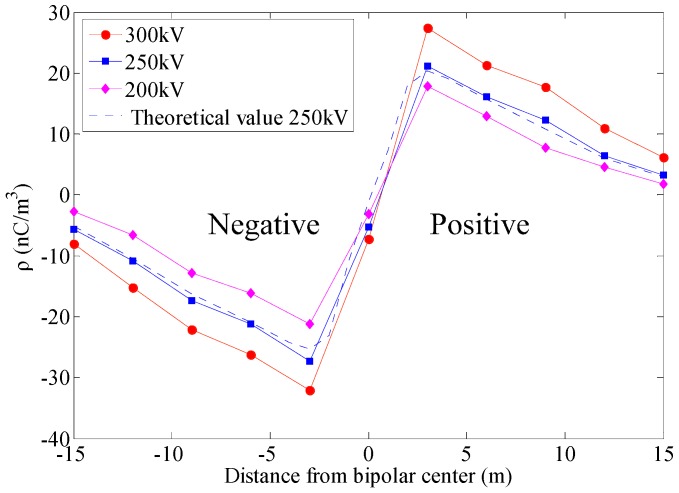
Lateral distribution test results of space charge density under different voltage level.

The voltage applied to the positive pole was 300 kV, 250 kV, and 200 kV. Correspondingly, the voltage applied to the negative pole was −300 kV, −250 kV and −200 kV. Other test conditions are the same as above. One of the measurement results is shown in Figure 18.

As the Figure 18 shown, with the increase of distance from the transmission lines, the space charge density value decreases and the maximum is located at the vertical projection of the transmission lines. The space charge density under UHVDC transmission lines is enhanced with the increase of the applied voltage level. The absolute value of the space charge density under the negative conductor is larger than the corresponding positive conductor. The test results of the space charge density basically consistent with the theoretical values, which demonstrate that the proposed wireless measurement system can accurately measure the space charge density under transmission lines.

Compared with previous studies such as the literatures [20,21,22,23], the conclusions from Figure 18, have the same change rule with them. Meanwhile, based on the wireless communication technologies and the experimental results the proposed sensor demonstrates that it can adapt to the complex electromagnetic environment under the UHVDC transmission line and can accomplish the accurate, flexible, and stable demands of the space charge density measurement.

## 6. Conclusions

In this paper, a wireless measurement system which is used to measure the distributed space charge density under UHVDC transmission lines for fulfilling the demands of flexibility, convenience and reliability has been developed. The measurement system which is proposed comprises many wireless sensors, data centers and servers as well as a base station. A space charge density sensor based on the ion counter method is presented. An ARM microprocessor and Zigbee RF module are applied to design the wireless sensor. The use of Digimesh networking protocol makes the WSN more flexible and increases the robustness in the complex UHVDC electromagnetic environment. The implementation and test results under UHVDC transmission lines demonstrate that the proposed wireless measurement system can adapt to the complex electromagnetic environment and accurately collect space charge density data.

## Figures and Tables

**Table 1:** 11 frequency channels of the RF module.

**Channel Number**	1	2	3	4	5	6	7	8	9	10	11
**Center Frequency/GHz**	2.410	2.415	2.420	2.425	2.430	2.435	2.440	2.445	2.450	2.455	2.460
